# Multivariate associations between psychiatric drug intake and grey matter volume changes in individuals at early stages of psychosis and depression

**DOI:** 10.1192/j.eurpsy.2024.573

**Published:** 2024-08-27

**Authors:** C. Weyer, D. Popovic, A. Ruef, L. Hahn, E. Sarişik, J. Fanning, J. Kambeitz, R. K. Salokangas, J. Hietala, A. Bertolino, S. Borgwardt, P. Brambilla, R. Upthegrove, S. J. Wood, R. Lencer, E. Meisenzahl, P. Falkai, N. Koutsouleris

**Affiliations:** ^1^Department of Psychiatry and Psychotherapy, LMU University Hospital, LMU Munich, Munich; ^2^Graduate School of Systemic Neurosciences, LMU Munich, Planegg-Martinsried; ^3^Max-Planck Institute of Psychiatry; ^4^International Max Planck Research School for Translational Psychiatry (IMPRS-TP), Munich; ^5^Department of Psychiatry and Psychotherapy, University of Cologne, Cologne, Germany; ^6^Department of Psychiatry, University of Turku, Turku, Finland; ^7^Department of Basic Medical Science, Neuroscience and Sense Organs, University of Bari Aldo Moro, Bari, Italy; ^8^Department of Psychiatry and Psychotherapy, University of Lübeck, Lübeck, Germany; ^9^University of Basel, Department of Psychiatry (Psychiatric University Hospital, UPK), Basel, Switzerland; ^10^Department of Neurosciences and Mental Health, Fondazione IRCCS Ca’ Granda Ospedale Maggiore Policlinico; ^11^Department of Pathophysiology and Transplantation, University of Milan, Milan, Italy; ^12^Institute of Mental Health, University of Birmingham; ^13^Early Intervention Service, Birmingham Women’s and Children’s NHS Foundation Trust; ^14^School of Psychology, University of Birmingham, Birmingham, United Kingdom; ^15^Centre for Youth Mental Health, University of Melbourne; ^16^Orygen, Melbourne, Australia; ^17^Institute for Translational Psychiatry, University Münster, Münster; ^18^Department of Psychiatry and Psychotherapy, Medical Faculty, Heinrich Heine University, Düsseldorf, Germany; ^19^Institute of Psychiatry, Psychology And Neurosciences, King’s College London, London, United Kingdom

## Abstract

**Introduction:**

Psychiatric drugs, including antipsychotics and antidepressants, are widely prescribed, even in young and adolescent populations at early or subthreshold disease stages. However, their impact on brain structure remains elusive. Elucidating the relationship between psychotropic medication and structural brain changes could enhance the understanding of the potential benefits and risks associated with such treatment.

**Objectives:**

Investigation of the associations between psychiatric drug intake and longitudinal grey matter volume (GMV) changes in a transdiagnostic sample of young individuals at early stages of psychosis or depression using an unbiased data-driven approach.

**Methods:**

The study sample comprised 247 participants (mean [SD] age = 25.06 [6.13] years, 50.61% male), consisting of young, minimally medicated individuals at clinical high-risk states for psychosis, individuals with recent-onset depression or psychosis, and healthy control individuals. Structural magnetic resonance imaging was used to obtain whole-brain voxel-wise GMV for all participants at two timepoints (mean [SD] time between scans = 11.15 [4.93] months). The multivariate sparse partial least squares (SPLS) algorithm (Monteiro *et al*. JNMEDT 2016; 271:182-194) was embedded in a nested cross-validation framework to identify parsimonious associations between the cumulative intake of psychiatric drugs, including commonly prescribed antipsychotics and antidepressants, and change in GMV between both timepoints, while additionally factoring in age, sex, and diagnosis. Furthermore, we correlated the retrieved SPLS results to personality domains (NEO-FFI) and childhood trauma (CTQ).

**Results:**

SPLS analysis revealed significant associations between the antipsychotic classes of benzamides, butyrophenones and thioxanthenes and longitudinal GMV decreases in cortical regions including the insula, posterior superior temporal sulcus as well as cingulate, postcentral, precentral, orbital and frontal gyri (Figure 1A-C). These brain regions corresponded most closely to the dorsal and ventral attention, somatomotor, salience and default network (Figure 1D). Furthermore, the medication signature was negatively associated with the personality domains extraversion, agreeableness and conscientiousness and positively associated with the CTQ domains emotional and physical neglect.

**Image:**

**Figure:**
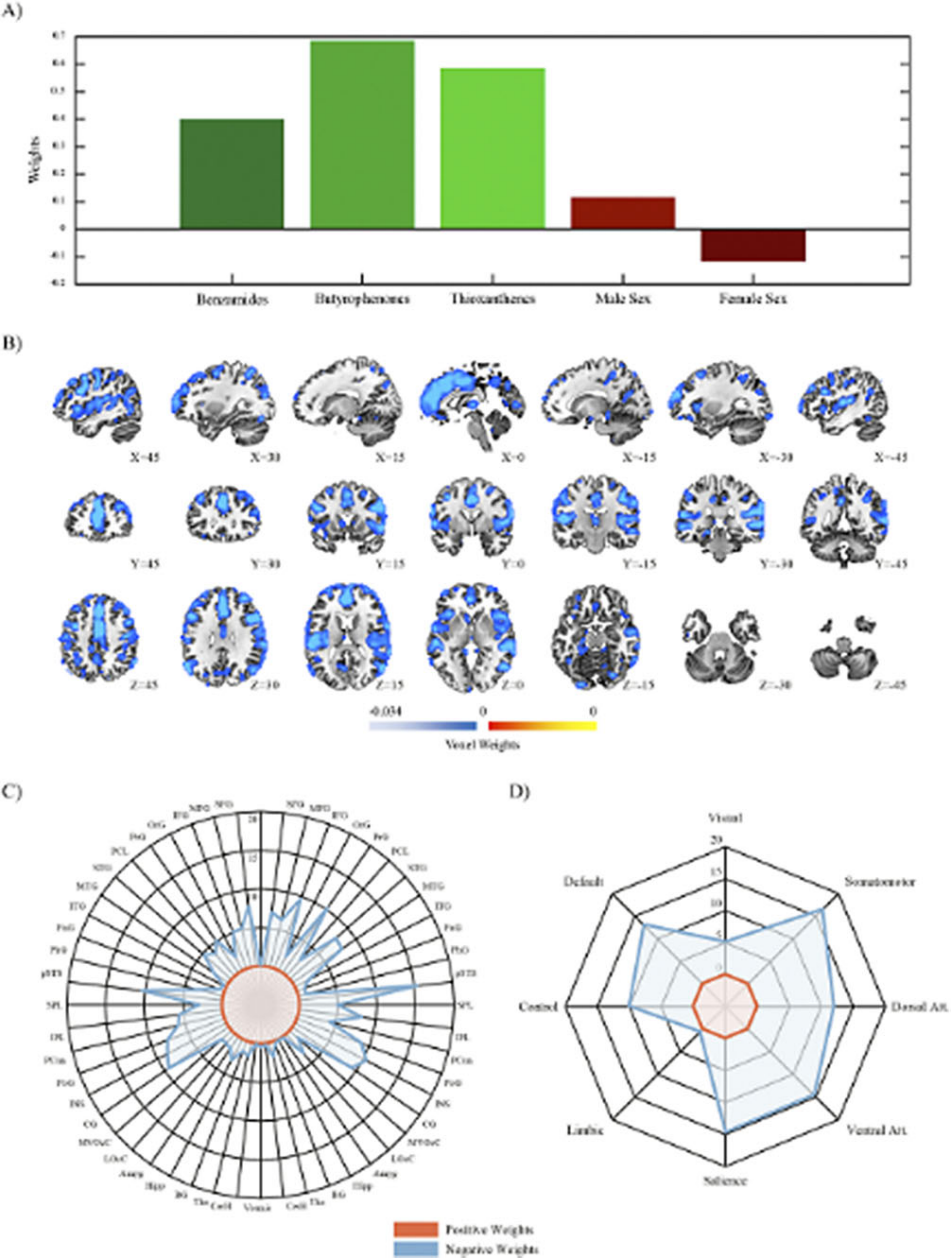

**Conclusions:**

Psychiatric drug intake over a period of one year was linked to distinct GMV reductions in key cortical hubs. These patterns were already visible in young individuals at early or subthreshold stages of mental illness and were further linked to childhood neglect and personality traits. Hence, a better and more in-depth understanding of the structural brain implications of medicating young and adolescent individuals might lead to more cautious, sustainable and targeted treatment strategies.

**Disclosure of Interest:**

None Declared

